# Investigation Into Shelf Life of Fresh Dates and Pistachios in a Package Modified With Nano-Silver

**DOI:** 10.5539/gjhs.v8n5p134

**Published:** 2015-09-17

**Authors:** Fateme Peyro Mousavi, Hasan Hashemi Pour, Amir Heidari Nasab, Ali A. Rajabalipour, Mohsen Barouni

**Affiliations:** 1 Department of Chemical Engineering, Faculty of Engineering, Islamic Azad University Science and Research Branch Tehran, Iran; 2 Kerman Shahid Bahonar University, Kerman, Iran; 3 Agricaltural Technical and Engineering Research Department, Agricultural and Natural Resource Center, Kerman, Iran; 4 Health Services Management Research Center, Institute for Futures Studies in Health, Kerman University of Medical Sciences, Kerman, Iran

**Keywords:** Mazafati dates, fresh pistachios, shelf life, silver nanoparticles, packages

## Abstract

**Aims:**

The aim of this study was to apply polymer films containing silver nanoparticles as a new method for increasing the shelf life and preserving the quality of export/commercial products of Kerman Province and determine the ideal temperature for preserving these products.

**Methods:**

After preparing nano-composite films containing silver nanoparticles (3% and 5% by weight), Mazafati dates were packed in them and stored with their control samples under four temperatures. In the second series, the films were filled with fresh pistachios and stored at four temperatures. In date samples, after 2, 7, 21 and 53 days of storing the samples were examined under the certified test of Iran Institute of Industrial Standard for Dates, which includes pH, TSS, acidity and reducing sugars tests. In pistachio samples the color values and market-friendly quality were evaluated after 1, 2, 3, 6, 7 and 8 days of storage.

**Results:**

In date samples, the pH value decreased with increasing acidity in 3 and 5 wt% of nano-silver and their control samples. In addition, in 5 wt% samples the acidity was higher than that in 3% samples, with pH being lower in the controls at almost all the intervals. Furthermore, pH values in 5% samples were higher in comparison with 3 wt% samples and controls. The amount of reducing sugars in the control samples was lower than those in 3 and 5 wt% samples. In relation to pistachio samples, the damage over time was greater in sample stored under higher temperatures.

**Conclusion:**

The maximum shelf life of the dates packaged in 5 wt% of silver nano-powder was 53 days and the best temperature to store samples was determined at 4°C. Packages containing nano-silver increased shelf life of fresh pistachios, with the best temperatures being 25°C and 0°C.

## 1. Introduction

Antimicrobial packaging is one of many applications of active packaging (Floros, Dock, & Han, 1997). Active packaging exhibits attributes beyond basic barrier properties, which are achieved by adding active ingredients to the packaging system and/or using actively functional polymers (Appendini & Hotchkiss, 2002). Antimicrobial packaging is able to kill or inhibit putrefying and pathogenic microorganisms that contaminate foods. The new antimicrobial function can be achieved by adding antimicrobial agents to the packaging system and/or using antimicrobial polymers that satisfy conventional packaging requirements. When the packaging system acquires antimicrobial activity, it limits or prevents microbial growth by extending the lag period and reducing the growth rate or decreasing live counts of microorganisms (Han, 2003). Compared to the goals of conventional food packaging such as (i) shelf life extension, (ii) quality maintenance and (iii) safety assurance, which could be achieved by various methods, antimicrobial packaging is specifically designed to control microorganisms that generally affect the above three goals adversely. Therefore some products, which are not sensitive to microbial spoilage or contamination, may not need the antimicrobial packaging system.

**Figure 1:**
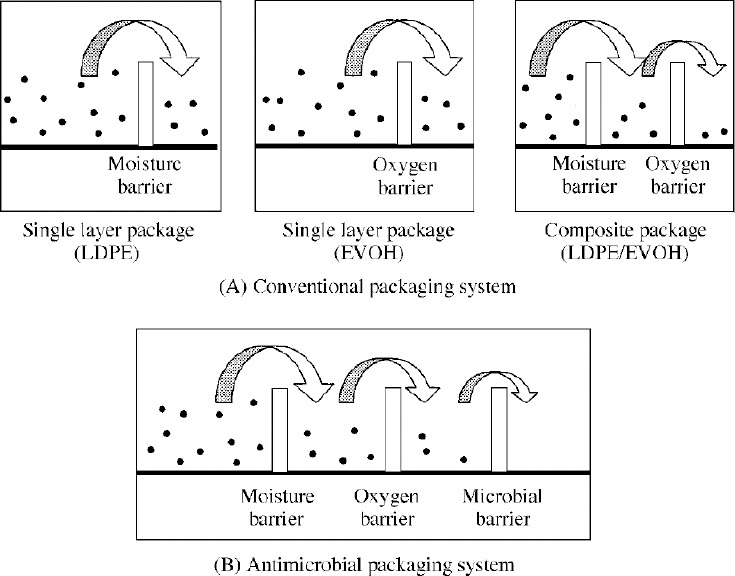
Hurdle technology in antimicrobial packaging system compared to the conventional packaging system. *Sources*: (Han, 2003)

The basic principle of these traditional preservation methods and antimicrobial packaging is the hurdle technology (Figure 1) (Han, 2003).

Silver is known as one of the antimicrobial agents. Silver is a safer anti-microbial agents in comparison with some organic anti-microbial agents that have been avoided because of the risk of their harmful effects on the human body. Silver has been described as being ‘oligo-dynamic’ because of its ability to confer a bactericidal effect to products containing silver, principally due to its anti-microbial activity and low toxicity to human cells. Its therapeutic property has been proven against a broad range of microorganisms, over 650 pathogenic organisms in the body even at low concentrations. The ability of silver to prevent bio-film formation has also been proven. A similar mechanism has been quoted for silver ions and nanoparticles. Silver nanoparticles are non-toxic and non-tolerant disinfectants. Use of silver nanoparticles leads to an increase in the number of particles per unit area and, thus, anti-bacterial effects can be maximized (Jia, Ma, Wei, Dong, & Qian, 2008). Therefore silver nanoparticles are able to partially protect foods with high humidity against microbial contamination (Sharma, Yngard, & Lin, 2009).

One of these highly consumed and useful products is Mazafati date. Dates with large amounts of vitamins, minerals and sugar are valuable products with a high nutritional value. Mazafati Routab is one of the commercial date varieties in Kerman Province (Iran); due to its high humidity it is spoiled and acetified under normal preservation conditions. Therefore this product is not yet able to find a good position in global markets. Dates like Mazafati Routab, which has more than 24% of moisture, are affected by yeasts and molds in hot and humid conditions and under the action of these microorganisms, fermentation (alcohol) and rancidity (production of lactic acid and acetic acid) take place.

The other highly consumed and useful product is fresh pistachio. Kerman (Iran) pistachio is one of the commercially important products. Pistachio is a non-oil export for bringing in foreign exchange every year (Pistachio export, 2012).

Today, after carpet, pistachio is one of the most important non-oil exports of Iran. Currently, 55% of production and 60% of exports of pistachio belong to Iran and currency revenues from pistachio exports will amount to more than $400 million in coming years.

Toxins produced by molds in pistachio produce aflatoxin contamination which is the main factor threatening pistachio exports (Gtalk, 2012). Therefore, if an appropriate method can be identified to control pistachio contamination, it will be possible to prevent premature deterioration of pistachio, creating the appearance of new market-friendly products suitable for export.

## 2. Materials Studied

### 2.1 Nano-Films

- Film-grade LDPE resin pellets (LF0200, MFI of 2 g/10 min, density of 0.92 g/mL, and softening point of 94°C) were directly mixed with each of the antimicrobial agents to produce 3 and 5 wt% nano-composite films, including P105 powder (a combination of 95% TiO_2_ powder which provided a base for doping of nano-silver, plus 5 wt% by weight of metal nano-silver with particle diameters of about 10 nm and a combination of 97% TiO_2_ powder plus 3 wt% by weight of metal nano-silver) separately and the mixture was fed into a twin-screw extruder machine with a screw diameter of 55 mm and a screw length/diameter ratio of 30 mm to be cut into master batch nano-granules. Proper amounts of master batch resins were then added to a single-screw blowing machine to fabricate the final nano-composite film (50 μm thick). These films were obtained from Pars Nanonasb Tehran, Iran.
- Mazafati date samples were procured from Bam Aziz Abad area (Kerman, Iran).
- Fresh pistachio samples were procured from orchards of Jannat Abad Sirjan (Kerman, Iran).

## 3. Methods and Techniques

### 3.1 Storage of Dates

Mazafati date samples procured from Aziz Abad, Bam (Kerman, Iran), were packed in both 3 and 5 wt% films and a control sample was taken for each package and enveloped in Penguin freezer bags. These bags were used because they are food-grade LDPE resins; the nano-film samples were also prepared from this resin and the profile given in the previous section. The packages with control samples were stored under four temperatures of 50°C, 20°C, 8°C, 4°C, and underwent testing to determine their quality 2, 7, 21 and 53 days after storage.

### 3.2 Storage of Fresh Pistachios

**Figure 2:**
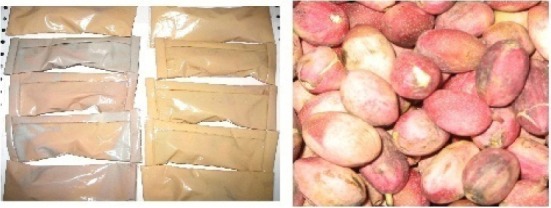
Samples packaged in 3 and 5 wt% covers of nano-films (A); Samples of fresh pistachios (B)

The samples were procured from fresh pistachio orchards of Jannat Abad Sirjan (Kerman, Iran) and packaged in 3 and 5 wt% of nano-films as shown in Figure 2. A control sample was prepared for each packaging by enveloping it in Penguin freezer bags as explained in the section on dates. The packages with control packets were stored under four temperature of 50°C, 25°C, 0°C, -18°C and 1, 2, 3, 6, 7 and 8 days after storage market-friendly quality and appearance were evaluated.

## 4. Results

### 4.1 Comparison of the Effects of Nanoparticles on Acidity of Sample Dates

**Figure 3:**
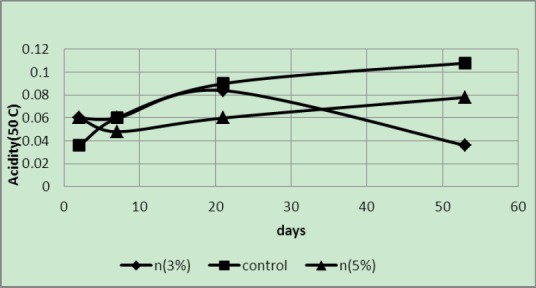
Acidity of samples packaged in covers with 3 and 5 wt% of silver nano-powder and the controls at a temperature of 50°C

Acidity was manifested at 50°C; however, the acidity of the control samples was less than the two samples in 3 and 5 wt% covers after some time in subsequent treatments. The pH value of the control sample was higher than the other two samples (Figure 3). The pH value of 5 wt% in less than 3 wt% of the sample increased. In sample 3 wt% reduction in acidity was observed on the last day, which is due to not ripening of this sample. This feature can be observed from low TSS of this sample on the 53th day (Table 1).

**Figure 4:**
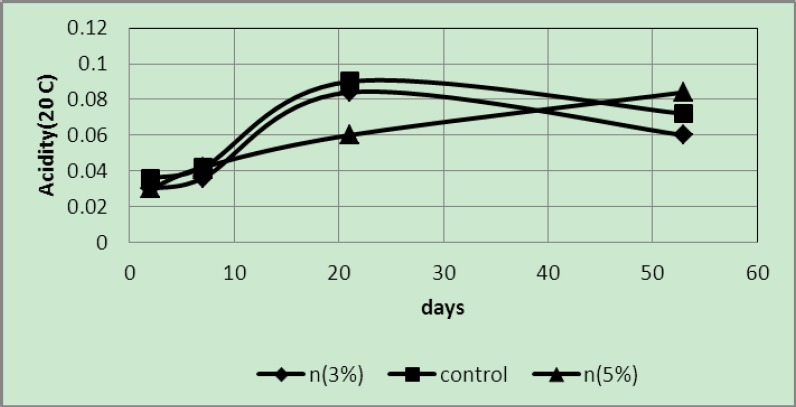
Acidity of samples packaged in covers with 3 and 5 wt% of silver nano-powder and the controls at a temperature of 20°C

**Table 1:** TSS values of 3 and 5 wt% samples and the controls under four storing temperatures

TSS	4°C			8°C			20°C			50°C		

Day\Percentage	3%	5%	control	3%	5%	control	3%	5%	control	3%	5%	control
**2**	9.2	10	9.8	10	6.9	9.3	10.2	8.3	10.4	10.6	10.4	10.3
**7**	9.4	7.9	10.4	8.5	9.2	12.1	10.6	10.3	8.3	10.4	8.8	7.5
**21**	13	8.9	11.8	7.9	8.3	9.2	11.4	9.6	9.1	7.8	4.2	8.7
**53**	8.8	8.5	8.8	9.5	8.7	7.8	6	8.3	8.5	7.7	6.1	9.4

The above graph shows that at 20°C the acidity of the control samples was higher than that of the samples packaged in covers with 3 and 5 wt% of silver nano-powder. In addition, variations of 3 wt% samples were similar to those of the control samples and higher than those in 5 wt% samples. Because of the differences between the samples in relation to ripening, an oscillatory mode can be seen in the figure.

**Figure 5:**
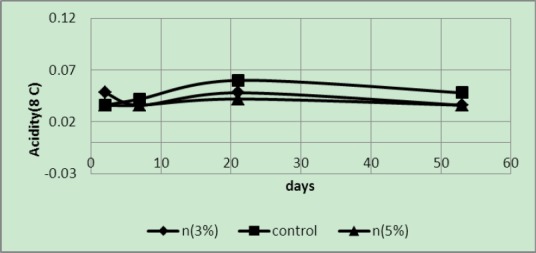
Acidity of samples packaged in covers with 3 and 5 wt% of silver nano-powder and the controls at a temperature of 8°C

Since the samples were stored under refrigerator temperatures the temperature rapidly decreased degraded samples; 5 wt% samples had the lowest acidity, with the controls exhibiting the highest acidity (Figure 5).

**Figure 6:**
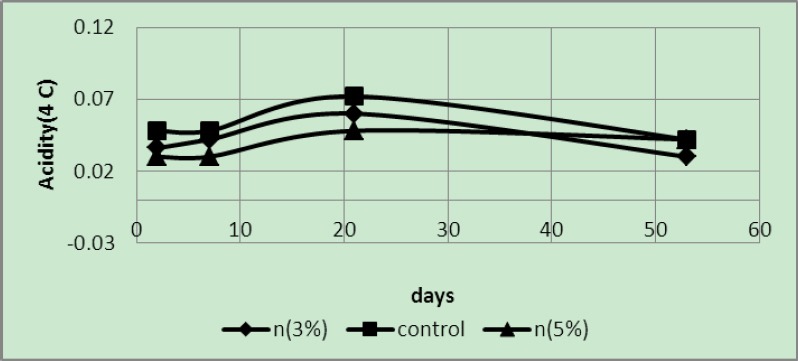
Acidity of samples packaged in covers with 3 and 5 wt% of silver nano-powder and the controls at a temperature of 4°C

As expected, at a temperature of 4°C (Figure 6) acidity of 5 wt% samples was less than that of 3 wt% samples and the control samples. It is noteworthy that samples treated in the fourth week exhibited less TSS than previous treatments; therefore, their acidity was less than the previous treatments (Table 1).

### 4.2 Comparison of the Effects of Nanoparticles on pH Values of the Sample Dates

**Figure 7:**
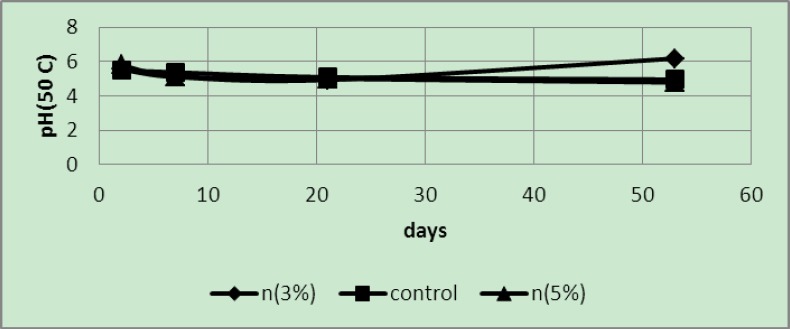
pH of samples packaged in covers with 3 and 5 wt% of silver nano-powder and the controls at a temperature of 50°C

The readings obtained from the pH meter showed that samples wrapped in sheets with 3 and 5 wt% of silver nanoparticles and their controls exhibited a regular decrease in pH values. Due to the very high temperatures and rapid degradation at a temperature of 50°C, pH values of the samples were close to each other (Figure 7).

**Figure 8:**
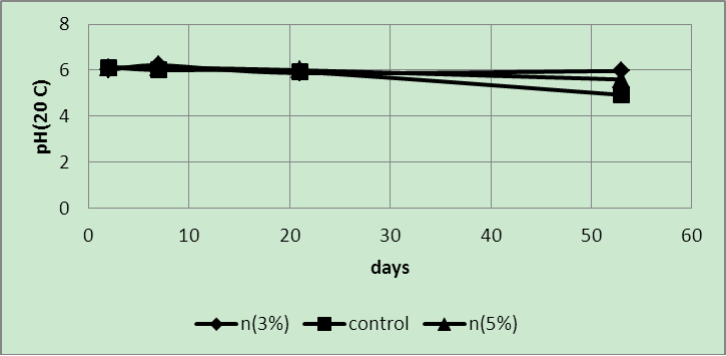
pH of samples packaged in covers with 3 and 5 wt% of silver nano-powder and the controls at a temperature of 20°C

In these samples (Figure 8), a regular decrease in pH values was seen, with a more rapid decline after 21 days. It was also implied that the pH values of the samples packaged in covers with 5 wt% nano-silver were a little higher than those in 3 wt% samples.

**Figure 9:**
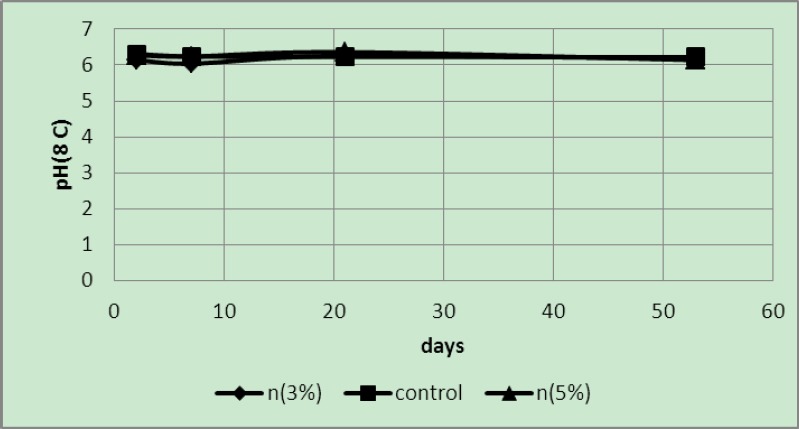
pH of samples packaged in covers with 3 and 5 wt% of silver nano-powder and the controls at a temperature of 8°C

It is probable that due to a low temperature almost no changes occurred in the pH values (Figure 9).

**Figure 10:**
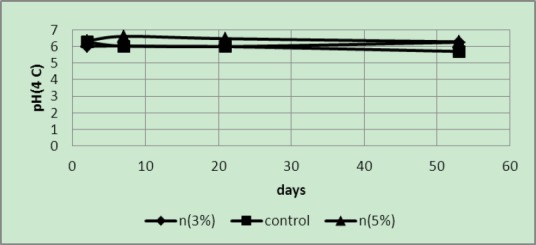
pH of samples packaged in covers with 3 and 5 wt% of silver nano-powder and the controls at a temperature of 4°C

The pH values of samples in covers with 5 wt% nano-silver were higher than the other samples and reduced over time to become acidic but generally the pH value of 5 wt% samples were higher than the controls and 3 wt% samples (Figure 10). At first, the pH values of 3 wt% samples were less than those of the controls but the pH values of the control samples rapidly decreased with time.

### 4.3 Comparison of the Effects of Nanoparticles in TSS and Reducing Sugars of Sample Dates

**Table 2:** Reducing sugars of 3 and 5 wt% samples and the controls under four storage temperatures

Reducing sugars	4°C			8°C			20°C			0°C		

Percent Day	3%	5%	control	3%	5%	control	3%	5%	control	3%	5%	control
**2**	0.554	0.693	0.362	0.64	0.462	0.438	0.52	0.438	0.489	0.594	0.52	0.594
**7**	0.462	0.462	0.438	0.693	0.416	0.378	1.387	0.396	0.438	0.756	0.489	0.693
**21**	1.387	0.924	0.924	0.924	0.924	0.594	1.04	1.04	0.756	0.756	0.489	1.04
**53**	1.188	1.664	1.188	1.04	1.188	0.832	1.188	0.924	0.64	0.554	0.756	0.462

### 4.4 Comparison of the Effects of Nanoparticles on the Appearance of Fresh Pistachios

**Figure 11:**
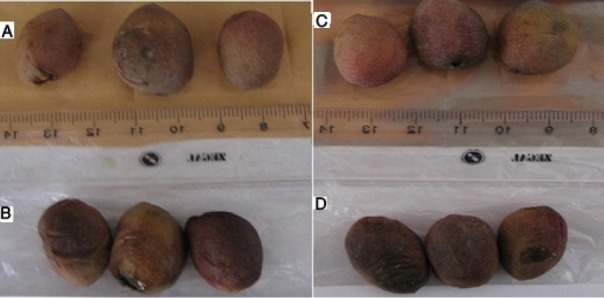
Samples placed under a temperature of 50°C on the second day. A) Envelope containing 3 wt% silver nano-powder. B) Control. C) Envelope containing 5 wt% silver nano-powder. D) Control

The color of the samples a day after storage under a temperature of 50°C changed from red to light brown but the final destruction occurred on the second day and the color changed to black (Figure 11). In almost all the cases more severe discoloration was observed in the control samples.

**Figure 12:**
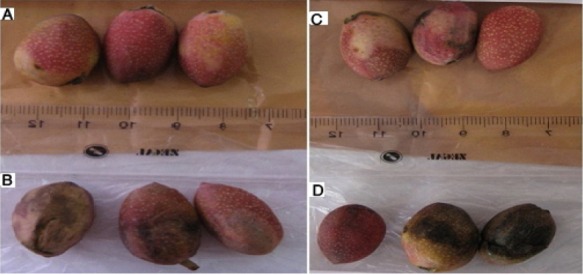
Samples placed at a temperature of 25°C on the third day. A) Envelope containing 3 wt% silver nano-powder. B) Control. C) Envelope containing 5 wt% silver nano-powder. D) Control.

In samples placed at a temperature of 25°C, there were not many changes on the first day. On the second day there were clear discolorations and on the third day there were black discolorations whereas the samples stored in the envelopes containing silver nano-powder exhibited less color changes (Figures 12).

**Figure 13:**
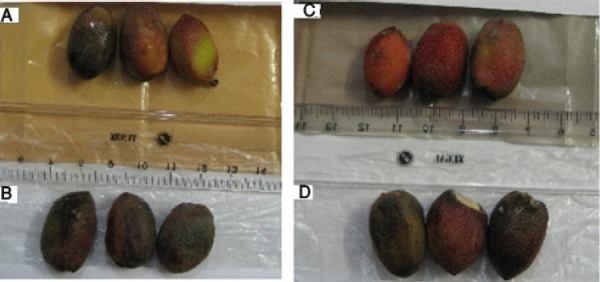
Samples placed at a temperature of 0°C on the eighth day. A) Envelope containing 3 wt% silver nano-powder. B) Control. C) Envelope containing 5 wt% silver nano-powder. D) Control.

Samples placed at a cold storage temperature of 0°C, almost at the freezing point, were edible for longer periods. The samples, especially the control samples on the eighth day, had lost their market-friendly quality (Figure 13).

**Figure 14:**
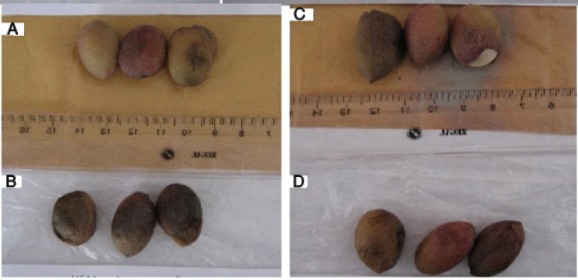
Samples placed at a temperature of -18°C on the eighth day. A) Envelope containing 3 wt% silver nano-powder. B) Control. C). Envelope containing 5 wt% silver nano-powder. D) Control

Samples placed at a cold temperature of -18°C were quite under freezing conditions. Therefore, apparently the color remained unchanged as long as they were under the freezing conditions. However, after leaving this condition, due to leaving the state of freezing lost Combining and color appearance were observed in the decay mode (Figure 14). As a result, storage at this temperature is not recommended.

## 5. Discussion

Generally, samples under a temperature of 50°C exhibited the highest and fastest degradation (spoiling) among the existing samples as the acidity of these samples was higher than samples at 20°C, 8°C and 4°C and therefore they had the lowest pH values. In addition, due to faster ripening rate and changes at this temperature, more severe fluctuations were seen in reducing sugars and TSS values as well as in the rate of pH changes.

As expected, changes at 20°C were higher than at the two other temperatures but in general, in all the cases, the pH of the samples in 5 wt% covers were less than those stored in 3 wt% covers and in the controls.

In short, 5 wt% samples exhibited less putrefaction than other samples. The pH values of the 3% samples were lower than those of the control samples, indicating that the 3 wt% samples underwent less putrefaction. Kubacka, using ethylene vinyl alcohol-containing nano-composite films made of silver-titanium dioxide, showed that these films have a high antimicrobial potential; however, similar studies on Mazafati Routab have not discovered yet and these experiments were probably the first one (Kubacka, Fernández-García, Cerrada, & Fernández-García, 2012).

The pH values at different temperatures showed that, except the values obtained at 50°C temperatures, in other temperatures as expected the pH values of 5 wt% samples were higher than others. The difference was quite obvious at 4°C; probably degradation in these samples took place very slowly due to the low temperature. Therefore no significant differences were found in the pH of the samples.

The pH values of 3 wt% samples and controls in most cases were close to each other. Based on a report by Fernandez, absorbent bags containing silver nanoparticles on packages of chicken can reduce microbial growth by 40%. It was observed that the samples in the nano-composite films exhibited higher pH values compared to samples in the pure LDPE films (control samples) (Fernández et al., 2009).

Covers containing 5 wt% nano-powder increased the shelf life of samples more than the covers containing 3 wt% silver nano-powder and the shelf life in both of these samples was higher than the controls. In addition, Emamifar stored samples of orange juice in the nano-composite films and showed that the shelf life of the orange juice samples in nano-composite films containing 5 wt% silver nano-powder increased nearly four folds (Emamifar, Kadivar, Shahedi, & Soleimanian-Zad, 2010).

The highest pH was seen at 21- and 53-day samples wrapped in covers containing 5 wt% silver nano-powder, indicating that at all the four temperatures, samples wrapped in covers containing 5 wt% silver nano-powder preserved their quality better than the other samples.

In fresh pistachios at a temperature of 50°C the final putrefaction occurred on the second day because of the high temperature. At a temperature of 25°C on the third day the control samples were black whereas the samples wrapped in covers containing silver nano-powder exhibited less color changes. A longer shelf life was observed in samples stored at 0°C, but they lost their market-friendly quality after eight days. The samples stored at a temperature of -18°C were under freezing conditions; therefore they lost Combining and color appearance so kept at this temperature is not recommended.

## 6. Conclusions

In this study, after storage of the date samples at four temperatures of 50°C, 20°C, 8°C and 4°C it was determined that the best storage temperature was 4°C for date samples, with the lowest levels of acidity at this temperature. In addition, the graphs showed that samples wrapped in covers containing 5 wt% silver nano-powder underwent less putrefaction than other samples, indicating that this amount of silver nano-powder results in an increase in the shelf life of date samples. Furthermore, the shelf life of 3 wt% samples was higher than the controls.

The best shelf life for covers containing 5 wt% silver nano-powder at 4°C temperature was 53 days, which means that after 53 days the samples still retained their quality and were edible.

On the other hand, this study showed that application of LDPE nano-composite packaging materials containing Ag nanoparticles is a new approach for preserving and extending the shelf life of fresh pistachios at four temperatures, especially at 25°C and 0°C. The quality of the packaging films, including good dispersion of nano-materials in the polymer matrix free from agglomeration, is very effective in the antimicrobial effects of these packaging materials.

Nano-silver exhibited a higher antimicrobial activity on yeasts and molds compared with other nanoparticles. It was also shown that application of this nano-packaging for storage of fresh pistachios was not sufficient for long-term storage.
